# Intratumoral Canine Distemper Virus Infection Inhibits Tumor Growth by Modulation of the Tumor Microenvironment in a Murine Xenograft Model of Canine Histiocytic Sarcoma

**DOI:** 10.3390/ijms22073578

**Published:** 2021-03-30

**Authors:** Federico Armando, Adnan Fayyad, Stefanie Arms, Yvonne Barthel, Dirk Schaudien, Karl Rohn, Matteo Gambini, Mara Sophie Lombardo, Andreas Beineke, Wolfgang Baumgärtner, Christina Puff

**Affiliations:** 1 Department of Pathology, University of Veterinary Medicine Hannover, Bünteweg 17, 30559 Hannover, Germany; federico.armando@tiho-hannover.de (F.A.); adnanf@najah.edu (A.F.); stefanie.arms@boehringer-ingelheim.com (S.A.); ybbarthel@aol.de (Y.B.); matteo.gambini@unimi.it (M.G.); or mara.lombardo@tiho-hannover.de (M.S.L.); andreas.beineke@tiho-hannover.de (A.B.); christina.puff@tiho-hannover.de (C.P.); 2 Department of Veterinary Medicine, An-Najah National University, Nablus 9720061, Palestine; 3 Fraunhofer Institute for Toxicology and Experimental Medicine, Nikolai-Fuchs-Straße 1, 30625 Hannover, Germany; dirk.schaudien@item.fraunhofer.de; 4 Institute for Biometry, Epidemiology and Information Processing, University of Veterinary Medicine Hannover, Bünteweg 2, 30559 Hannover, Germany; karl.rohn@tiho-hannover.de; 5 Dipartimento di Medicina Veterinaria (DIMEVET), Università degli Studi di Milano, Via dell’Università 6, 26900 Lodi, Italy

**Keywords:** canine distemper virus, canine histiocytic sarcoma, microvessel density, murine xenograft model, oncolytic virus, tumor microenvironment

## Abstract

Histiocytic sarcomas refer to highly aggressive tumors with a poor prognosis that respond poorly to conventional treatment approaches. Oncolytic viruses, which have gained significant traction as a cancer therapy in recent decades, represent a promising option for treating histiocytic sarcomas through their replication and/or by modulating the tumor microenvironment. The live attenuated canine distemper virus (CDV) vaccine strain Onderstepoort represents an attractive candidate for oncolytic viral therapy. In the present study, oncolytic virotherapy with CDV was used to investigate the impact of this virus infection on tumor cell growth through direct oncolytic effects or by virus-mediated modulation of the tumor microenvironment with special emphasis on angiogenesis, expression of selected MMPs and TIMP-1 and tumor-associated macrophages in a murine xenograft model of canine histiocytic sarcoma. Treatment of mice with xenotransplanted canine histiocytic sarcomas using CDV induced overt retardation in tumor progression accompanied by necrosis of neoplastic cells, increased numbers of intratumoral macrophages, reduced angiogenesis and modulation of the expression of MMPs and TIMP-1. The present data suggest that CDV inhibits tumor growth in a multifactorial way, including direct cell lysis and reduction of angiogenesis and modulation of MMPs and their inhibitor TIMP-1, providing further support for the concept of its role in oncolytic therapies.

## 1. Introduction

Neoplastic diseases represent one of the leading causes of death in humans and small animals [1,2,3]. Despite the frequency of tumors, adequate, efficient therapies are often missing and commonly provide only a limited increase in life expectancy and disease-free intervals [4,5,6,7]. Neoplasms with a poor prognosis despite several treatment strategies, including radio- and chemotherapy in humans and dogs, include histiocytic sarcomas [7,8]. These tumors rarely occur in humans but have a high incidence in dogs and breed predisposition for Bernese mountain dogs and flat-coated retrievers in several studies [9,10,11]. Canine histiocytic sarcomas comprise localized and disseminated forms [12]. The median survival time in dogs suffering from the disseminated variant ranges between 43 days and 106 days [11,13]. This highlights the need for additional therapeutic approaches complementing or replacing conventional treatment schemes.

An interesting approach, coming into focus over the past years, is represented by viral oncolysis. The first incidental and anecdotal descriptions of tumor regressions after a spontaneous virus infection or vaccination date several decades back [14]. Since then, viruses of multiple families, such as Adenoviridae, Herpesviridae, Paramyxoviridae, Poxviridae and Reoviridae, have been intensively studied in various human tumors, often using vaccine or genetically modified virus strains [15,16,17,18]. Compared to human medicine, different viruses are considered as potential oncolytic agents in veterinary medicine, with the most promising candidates belonging to Poxviridae, Adenoviridae and Paramyxoviridae [18,19,20].

Initially, tumor regression induced by oncolytic viruses was thought to occur through direct lysis of tumor cells [21]. However, recent evidence indicates that oncolytic viruses can also lead to tumor regression through other mechanisms, such as altering the tumor microenvironment into a milieu that enhances anticancer activity by modulating the immune system and inhibiting angiogenesis [22,23,24,25,26]. Another promising therapeutic approach is the use of oncolytic viruses for targeting matrix metalloproteinases (MMPs) and their inhibitors (TIMPs), which have long been associated with tumor progression [27].

Canine distemper virus (CDV), a member of the family Paramyxoviridae, genus Morbillivirus, represents an interesting candidate for viral oncolysis [18]. Similarities between CDV and measles (MV) and its ability to infect a wide variety of different cell types, including hematopoietic cells, together with an excellent safety record, make this virus an attractive candidate for oncolytic viral therapy [28,29]. Apart from its excellent safety profile and similarities with MV, preliminary studies revealed promising results in the ability of CDV to selectively infect and lyse canine hematopoietic tumor cell lines, including canine histiocytic sarcoma cells (CCT and DH82 cells) and neoplastic lymphocytes isolated from dogs with B and T cell lymphomas [28,30,31]. In addition, an in vitro study showed that CDV infection of DH82 cells leads to modulation of important regulators of the extracellular matrix metabolism, matrix metalloproteinases and their inhibitors, pointing towards a less malignant phenotype of infected tumor cells [31].

Results from a previously established murine xenotransplantation model of canine histiocytic sarcomas clearly indicated that expression of MMP-2, MMP-9, MMP-14 and TIMP-1 by tumor cells and tumor-associated macrophages and the positive correlation between MMP expression and microvessel density contributed to tumor progression and invasion [32]. Moreover, transplantation of persistently CDV infected DH82 cells resulted in a reduction of tumor cell migration in vitro [33] and results in complete spontaneous tumor regression in a murine xenograft model [34]. In the present study, a more clinically relevant intratumoral application of CDV has been evaluated in a previously established murine xenotransplantation model of canine histiocytic sarcoma. Thus, the hypothesis of this study was that a CDV infection leads to a regression of canine histiocytic sarcomas in vivo. To test this hypothesis, the aims of the present report were (i) to determine whether CDV infection of tumor cells can be maintained during in vivo treatment of established tumors and (ii) to investigate the impact of a CDV infection on tumor progression through direct oncolytic effects or modulation of the tumor microenvironment with special emphasis on tumor-associated macrophages, expression of selected MMPs and TIMP-1, and angiogenesis.

## 2. Results

### 2.1. A Tenfold Intratumoral Infection Leads to a Significantly Reduced Tumor Growth Compared to Controls

**Figure 1:**
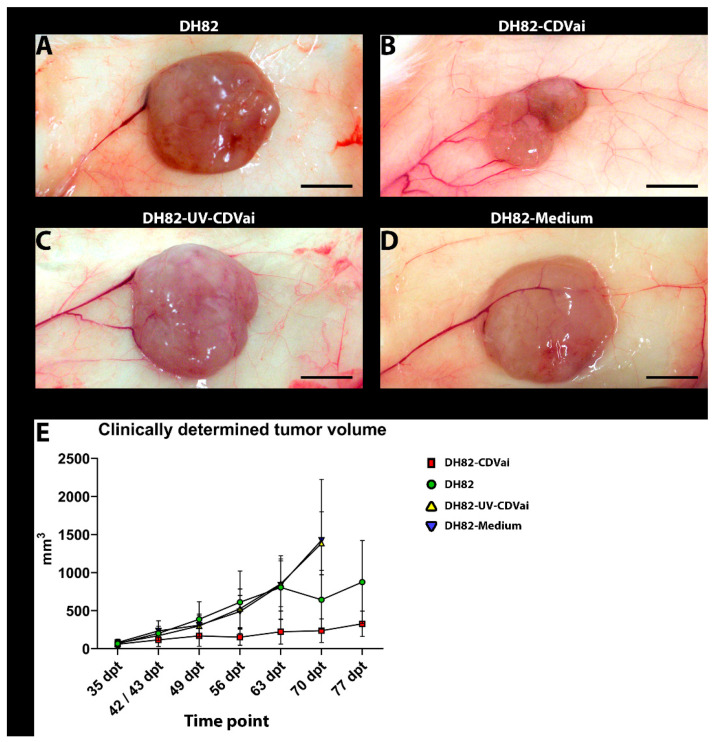
Representative gross pictures of xenografts at 63 dpt of (**A**) non-infected DH82 cells (DH82), (**B**) DH82 cells infected with canine distemper virus (CDV) (DH82-CDVai), (**C**) DH82 cells injected with UV-inactivated CDV (DH82-UV-CDVai) and (**D**) DH82 cells injected with medium (DH82-medium). DH82-CDVai neoplasms were significantly smaller than tumors of all control groups. (**A**–**D**) Scale Bar = 0.5 cm, (**E**) Graphical overview of tumor growth (mm^3^) over time (days post-transplantation, dpt). Interestingly, the volume of DH82-CDVai xenografts remains relatively stable, while controls showed a continuous progression.

Subcutaneously xenotransplanted DH82 cells formed tumors with a diameter of approximately 0.5 cm 35 days post-transplantation (dpt), displaying no significant differences between the groups. At this time point, treatment by intratumoral infection with CDV was initiated and repeated 10 times every second day and compared to controls. The latter comprised groups receiving either an intratumoral application of UV-inactivated CDV or cell culture medium or were left untreated. Animals receiving intratumoral injections with UV-inactivated CDV or medium were preliminary sacrificed at 70 dpt because tumors exceeded a volume of 1.6875 cm^3^ (~1.5 cm in diameter). Therefore, the last planned time point (77 dpt) was excluded from further analyses. The clinically determined tumor size is shown in Figure 1.

Over time, all groups displayed a significant increase (*p* values ranging from 0.0002 to 0.0303) in tumor volume between 35 dpt and the day of death.

Interestingly, the volume of tumors infected with CDV was already significantly smaller at 37 dpt when compared to neoplasms injected with medium (*p* = 0.0058). At 41 dpt non-treated neoplasms (*p* = 0.0006) and tumors of both intratumorally treated control groups exhibited a significantly larger size (*p* = 0.0040 and *p* = 0.0006) than neoplasms infected with CDV. Similar findings were present over time with few exceptions at 44 dpt and 54 dpt. In the former time point (44 dpt), no significant difference was observed between the CDV-infected group and the group treated with UV-inactivated CDV or left untreated. At the latter time point (54 dpt), only untreated control tumors were significantly larger than neoplasms infected with CDV (*p* = 0.0306). Unfortunately, the size of both intratumorally injected control groups (DH82-UV-CDVai and DH82-medium) was not determined at this time point. Starting at 65 dpt, no significant differences were found between CDV-infected and medium-injected neoplasms and untreated controls. Tumors injected with UV-inactivated CDV were significantly larger (*p* = 0.0225) than neoplasms infected with CDV until 70 dpt at which animals treated with UV-inactivated virus were sacrificed. Taken together, a tenfold intratumoral injection with CDV-Ond led to a significant reduction in tumor growth from day 35 to day 70 compared to controls.

### 2.2. Murine Xenotransplantation Model of Canine Histiocytic Sarcoma Was Successfully Confirmed

All animals that received subcutaneous DH82 cell xenotransplantation developed a round to oval nodular mass at the injection site. On histological examination, neoplasms were composed of poorly differentiated tumor cells forming infiltratively growing, nonencapsulated, cell-rich masses. Neoplastic cells were round to oval with varying sizes (20–60 μm) and distinct cell boundaries. In addition, the cells showed homogenous to finely granulated or vacuolated, eosinophilic cytoplasm and oval to polygonal, central to eccentric nuclei with coarse chromatin and prominent eosinophilic nucleoli. A moderate to high anisocytosis and anisokaryosis was present. The mitotic index varied from 8–11 mitotic figures per high-power field. Tumors from all groups showed a mild multifocal infiltration with mononuclear cells. In addition to HE-stained sections, tumor cells were highlighted using immunohistochemical staining of neoplastic cells with a CD44 antibody. Tumors of all groups displayed a strong CD44 expression within neoplastic cells.

### 2.3. Acute CDV-Infection Leads to a Growth Retardation of Xenotransplants Accompanied by Necrosis in a Murine Model of Canine Histiocytic Sarcoma

Areas of necrosis were detected in all groups, which appeared as hypereosinophilic areas with loss of cellular details and indistinct cell boundaries in addition to nuclear pyknosis, karyorrhexis and karyolysis.

**Figure 2:**
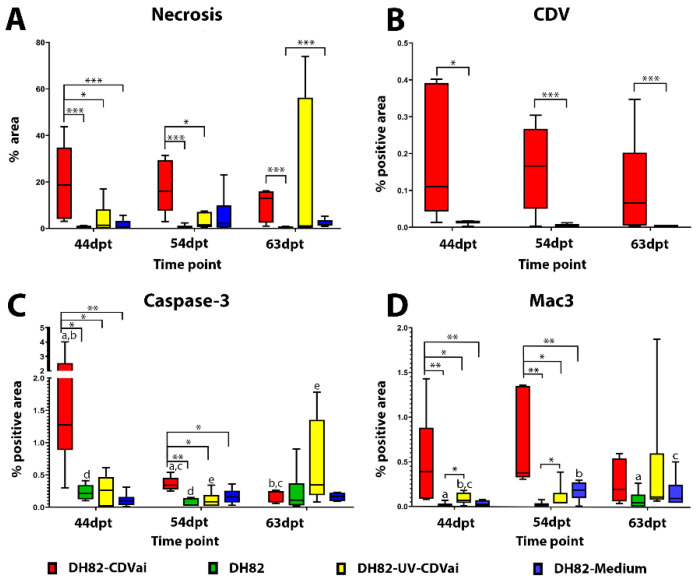
(**A**) Comparative illustration of the percentage of necrotic tumor tissue in non-infected (DH82), intratumorally CDV-infected (DH82-CDVai), intratumorally UV-inactivated CDV-injected (DH82-UV-CDVai) and intratumorally medium-injected (DH82-medium) xenografts. (**B**) The graph shows the intratumoral CDV-positive area in DH82-CDVai and DH82-UV-CDVai xenografts. (**C**) Apoptotic rate as determined by cleaved caspase 3 staining is low in all groups. (**D**) Intratumoral macrophage infiltration as determined by Mac3 immunolabeling. All graphs represent Box and whisker plots with statistically significant differences between different groups at the same time point (* *p* < 0.05, ** *p* ≤ 0.01 and *** *p* ≤ 0.001) and between different time points within the same group (a–e; *p* < 0.05).

DH82-CDVai xenografts showed a higher median area of necrosis compared to non-infected controls at all time points (Supplementary Table S1). Statistical analysis (Figure 2A) of the results revealed significantly larger necrosis areas in DH82-CDVai xenografts compared with all control groups at 44 dpt (*p* values between 0.0051 and 0.0306). At 54 dpt DH82-CDVai xenografts displayed significantly larger areas of necrosis than non-treated DH82 neoplasms (*p* = 0.0075) or DH82-UV-CDVai tumors (*p* = 0.0225). Areas of necrosis were significantly larger in DH82-CDVai xenografts than non-treated DH82 transplants (*p* = 0.0081) at 63 dpt. At 44 and 54 dpt, no significant differences were observed between the control groups. At 63 dpt, DH82-medium xenografts displayed significantly larger necrosis areas than non-treated DH82 neoplasms (*p* = 0.0082), whereas no significant differences were observed between the other control groups.

In none of the groups, statistically significant changes over time were observed. All data are summarized in Supplementary Table S1.

### 2.4. CDV-Nucleoprotein Was Detected in Intratumorally Treated DH82 Cell Xenografts

CDV nucleoprotein was detected in DH82-CDVai and DH82-UV-CDVai xenografts using immunohistochemistry. Immunoreactivity was characterized by a localized intracytoplasmic or membranous staining within tumor cells. Morphometric analysis of DH82-CDVai xenografts exhibited a median positive area of 0.126% at 44 dpt (after 5× intratumoral infection with CDV), 0.227% at 54 dpt (after 10× intratumoral infection with CDV), and 0.121% at 63 dpt (10 days after last treatment). DH82-UV-CDVai xenografts exhibited a median positive area of 0.014% at 44 dpt (after 5× intratumoral injection with UV-inactivated CDV), 0.003% at 54 dpt (after 10× times intratumoral injection with UV-inactivated CDV), and 0.002% at 63 dpt (10 days after last intratumoral injection with UV-inactivated CDV). Although the replication of the virus was limited to a small percentage of area in both investigated groups, CDV immunoreactivity was present in larger areas in DH82-CDVai compared to DH82-UV-CDVai at all time points investigated (*p* values between 0.0081 and 0.0200; Figure 2B). All data are reported in Supplementary Table S1.

### 2.5. Acute CDV-Infection Leads to an Enhanced Apoptosis in DH82 Cell Xenografts at Early Time Points

**Figure 3:**
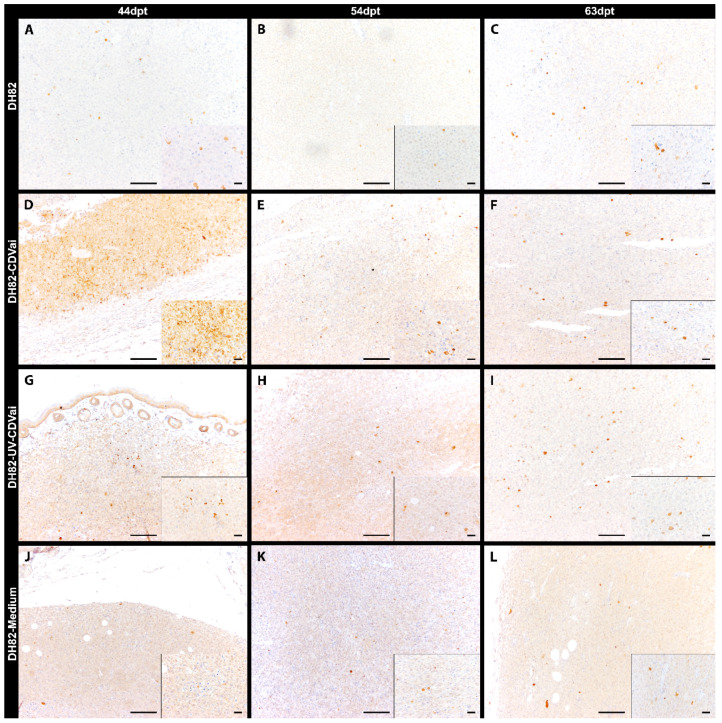
Cleaved caspase-3 immunolabeling in murine subcutaneous DH82 cell xenografts (**A**–**L**). (**A**–**C**) display non-infected (DH82), (**D**–**F**) intratumorally CDV-infected (DH82-CDVai), (**G**–**I**) intratumorally UV-inactivated CDV-injected (DH82-UV-CDVai) and (**J**–**L**) intratumorally medium-injected (DH82-medium) neoplasms at (**A**,**D**,**G**,**J**) 44 days post-transplantation (dpt), (**B**,**E**,**H**,**K**) 54 dpt, and (**C**,**F**,**I**,**L**) 63 dpt. Scale bar = 100 µm and scale bar—insert = 20 µm.

Apoptotic tumor cells were detected by measuring the area exhibiting cleaved caspase-3 immunoreactivity (Figure 3). In general, only small areas expressed cleaved caspase-3 in all groups, with the largest area (1.27%) present in DH82-CDVai at 44 dpt. (Figure 2C). However, DH82-CDVai xenografts displayed significantly larger cleaved caspase-3 antigen-positive areas at 44 dpt and 54 dpt than control groups of non-infected DH82, DH82-UV-CDVai and DH82-medium xenografts (*p* values ranging from 0.0081 to 0.0358), whereas no significant differences were observed between the different control groups. At 63 dpt, no significant differences were found between the different groups. The cleaved caspase-3 expressing area decreased significantly in DH82-CDVai over time (*p* values ranging from 0.0081 to 0.0358). In non-infected DH82 transplants, the cleaved caspase-3 immunopositive area was significantly smaller (*p* = 0.0306) at 54 dpt compared to 44 dpt. Furthermore, there was an increase in the cleaved caspase-3 immunopositive area at 63 dpt in DH82-UV-CDVai xenografts compared to tumors at 54 dpt in this group (*p* = 0.0453). DH82-medium xenotransplants displayed no changes in the cleaved caspase-3 immunopositive area over time. All data are summarized in Supplementary Table S1. Summarized, the results revealed that, despite changes between individual groups, apoptosis was only present in a small area, while the area of necrotic cells differed markedly between tumors infected with CDV and controls.

### 2.6. Acute Intratumoral CDV-Infection Is Associated with Increased Numbers of Tumor-Associated Macrophages in DH82 Cell Xenografts

**Figure 4:**
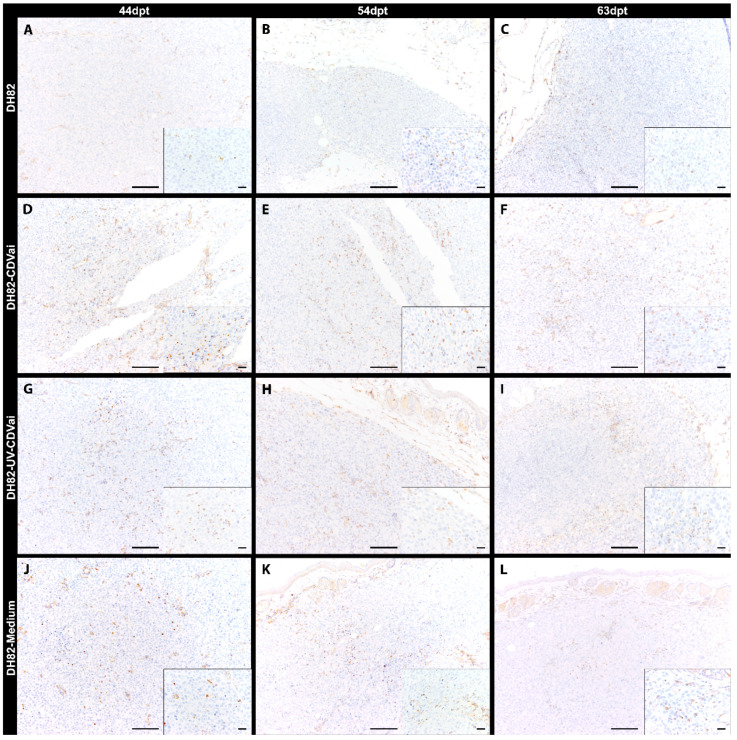
Mac3 immunolabeling for assessment of tumor-associated macrophages in murine subcutaneous DH82 cell xenografts (**A**–**L**). (**A**–**C**) display non-infected (DH82), (**D**–**F**) intratumorally canine distemper virus (CDV)-infected (DH82-CDVai), (**G**–**I**) intratumorally UV-inactivated CDV-injected (DH82-UV-CDVai) and (**J**–**L**) intratumorally medium-injected (DH82-medium) neoplasms at (**A**,**D**,**G**,**J**) 44 days post-transplantation (dpt), (**B**,**E**,**H**,**K**) 54 dpt and (**C**,**F**,**I**,**L**) 63 dpt. Scale bar = 100 µm and scale bar—insert = 20 µm.

Tumor-associated macrophages were investigated by determining the Mac3 (CD107b/LAMP2) immunoreactive area (Figure 4). A mild to moderate infiltration with Mac3 expressing murine macrophages was evident in central tumor areas and around necrotic foci in DH82-CDVai and all control groups. Interestingly, DH82-CDVai xenografts exhibited a significantly larger Mac3 immunoreactive area at 44 dpt and 54 dpt than all control groups (*p* values ranging from 0.0051 to 0.0453; Figure 2D). In addition, significantly larger Mac3 exhibiting areas were present in DH82-UV-CDVai than in non-treated controls at 44 dpt and 54 dpt (*p* = 0.0306). At 63 dpt, no significant differences were observed between the different groups.

In non-infected DH82 xenotransplants, a significant increase in the Mac3-positive area was present from 44 dpt to 63 dpt (*p* = 0.0453). Furthermore, a significant increase between 44 dpt and 54 dpt (*p* = 0.0306) or 63 dpt (*p* = 0.0306), respectively, was found in DH82-medium transplants. In all other groups, no significant changes were present over time. All data are summarized in Supplementary Table S1.

### 2.7. Acute CDV-Infection Leads to a Modulation of the MMPs and TIMP-1 Expression in DH82 Cell Xenografts

**Figure 5:**
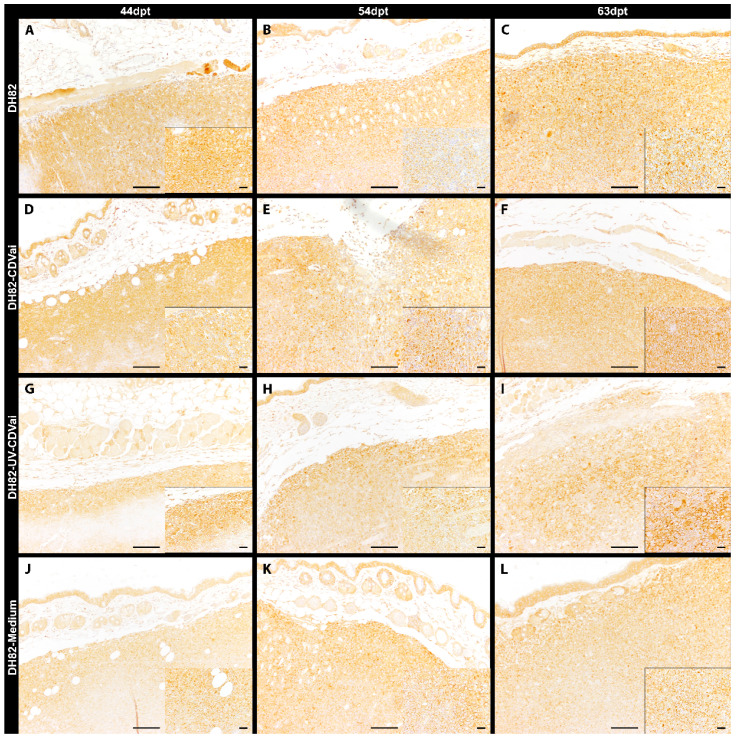
Peripheral MMP-2 immunolabeling in murine subcutaneous DH82 cell xenografts (**A**–**L**). (**A**–**C**) display non-infected (DH82), (**D**–**F**) intratumorally canine distemper virus (CDV)-infected (DH82-CDVai), (**G**–**I**) intratumorally UV-inactivated CDV-injected (DH82-UV-CDVai) and (**J**–**L**) intratumorally medium-injected (DH82-medium) neoplasms at (**A**,**D**,**G**,**J**) 44 days post-transplantation (dpt), (**B**,**E**,**H**,**K**) 54 dpt and (**C**,**F**,**I**,**L**) 63 dpt. Scale bar = 100 µm and scale bar—insert = 20 µm.

**Figure 6:**
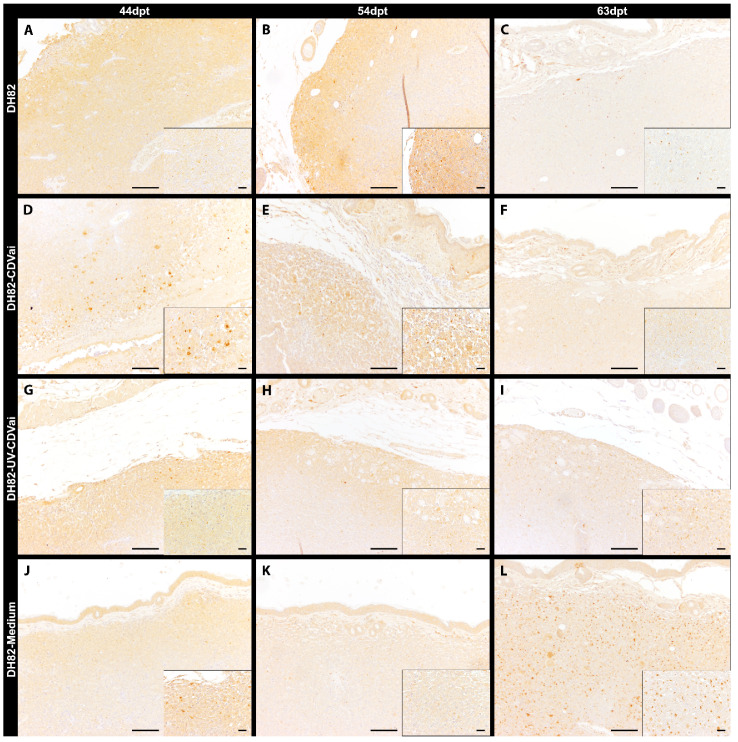
Peripheral MMP-9 immunolabeling in murine subcutaneous DH82 cell xenografts (**A**–**L**). (**A**–**C**) display non-infected (DH82), (**D**–**F**) intratumorally canine distemper virus (CDV)-infected (DH82-CDVai), (**G**–**I**) intratumorally UV-inactivated CDV-injected (DH82-UV-CDVai) and (**J**–**L**) intratumorally medium-injected (DH82-medium) neoplasms at (**A**,**D**,**G**,**J**) 44 days post-transplantation (dpt), (**B**,**E**,**H**,**K**) 54 dpt and (**C**,**F**,**I**,**L**) 63 dpt. Scale bar = 100 µm and scale bar—insert = 20 µm.

**Figure 7:**
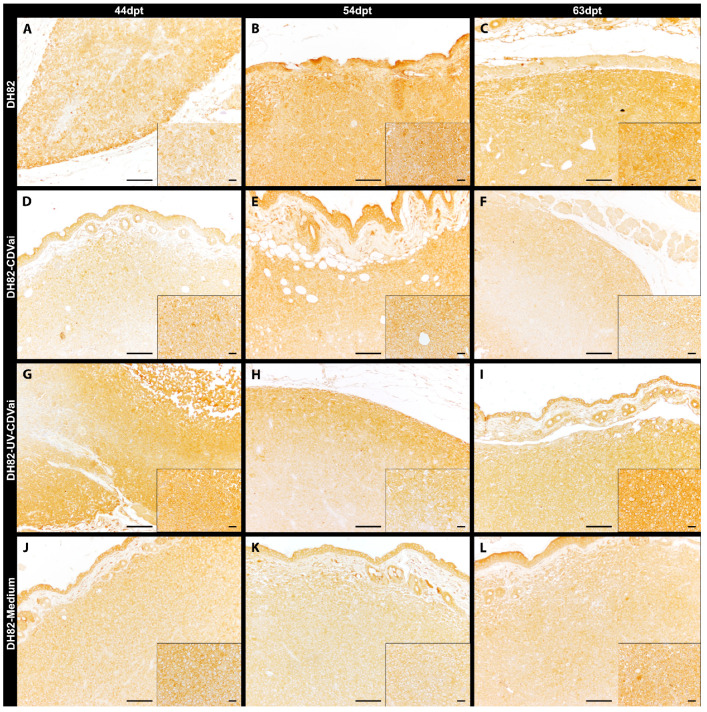
Peripheral MMP-14 immunolabeling in murine subcutaneous DH82 cell xenografts (**A**–**L**). (**A**–**C**) display non-infected (DH82), (**D**–**F**) intratumorally canine distemper virus (CDV)-infected (DH82-CDVai), (**G**–**I**) intratumorally UV-inactivated CDV-injected (DH82-UV-CDVai) and (**J**–**L**) intratumorally medium-injected (DH82-medium) neoplasms at (**A**,**D**,**G**,**J**) 44 days post-transplantation (dpt), (**B**,**E**,**H**,**K**) 54 dpt and (**C**,**F**,**I**,**L**) 63 dpt. Scale bar = 100 µm and scale bar—insert = 20 µm.

**Figure 8:**
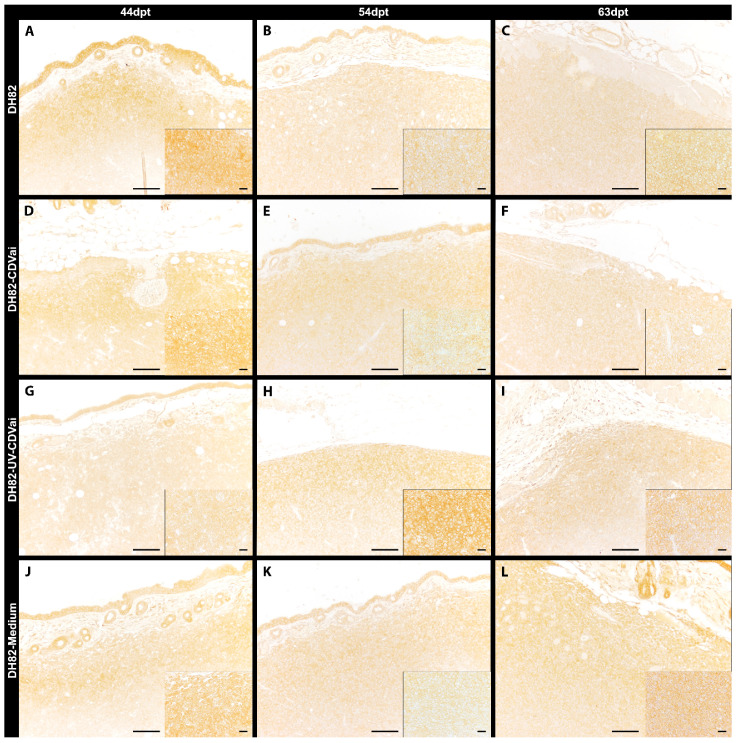
Peripheral TIMP-1 immunolabeling in murine subcutaneous DH82 cell xenografts (**A**–**L**). (**A**–**C**) display non-infected (DH82), (**D**–**F**) intratumorally canine distemper virus (CDV)-infected (DH82-CDVai), (**G**–**I**) intratumorally UV-inactivated CDV-injected (DH82-UV-CDVai) and (**J**–**L**) intratumorally medium-injected (DH82-medium) neoplasms at (**A**,**D**,**G**,**J**) 44 days post-transplantation (dpt), (**B**,**E**,**H**,**K**) 54 dpt and (**C**,**F**,**I**,**L**) 63 dpt. Scale bar = 100 µm and scale bar—insert = 20 µm.

All neoplasms expressed MMP-2, MMP-9, MMP-14 and TIMP-1 independent of the infection state but displayed a variable distribution and intensity as demonstrated by immunohistochemistry (Figure 5, Figure 6, Figure 7 and Figure 8).

**Figure 9:**
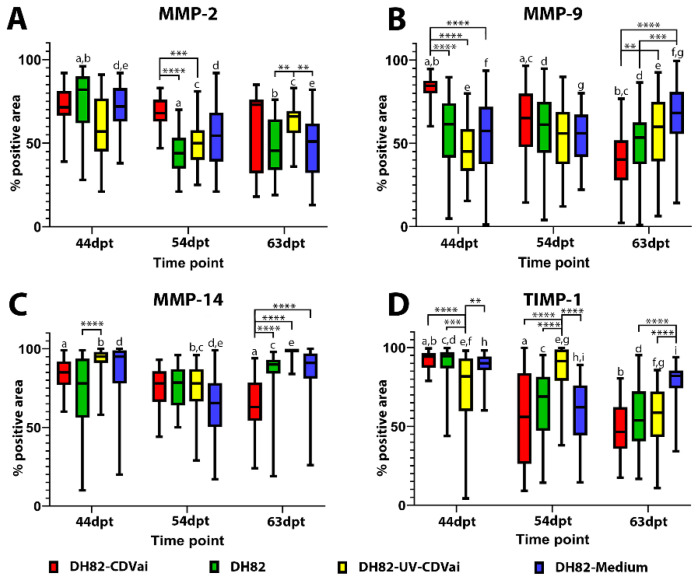
Comparative illustration of the percentage of immunolabeled tumor tissue within the periphery of non-infected (DH82), intratumorally canine distemper virus (CDV)-infected (DH82-CDVai), intratumorally UV-inactivated CDV-injected (DH82-UV-CDVai) and intratumorally medium-injected (DH82-medium) xenografts. The graphs show the percentage of (**A**) matrix metalloproteinase (MMP)-2, (**B**) MMP-9, (**C**) MMP-14 and (**D**) their inhibitor (TIMP)-1 immunolabeling over time (days post-transplantation, dpt). All graphs represent Box and whisker plots with statistically significant differences between different groups at the same time point (** *p* ≤ 0.01, *** *p* ≤ 0.001, and **** *p* ≤ 0.0001) and between different time points within the same group (a–i; *p* < 0.05).

The immunopositive area for MMP-2 (Figure 5), MMP-9 (Figure 6), MMP-14 (Figure 7), and TIMP-1 (Figure 8) was in all groups, at most of the time points, more prominent in the periphery compared to central localizations (*p* values ranging from <0.0001 to 0.0003). The only exceptions were present at 63 dpt, where MMP-2 in DH82-CDVai and MMP-14 in DH82-CDVai, and DH82-UV-CDVai lacked significant differences between the different localizations. Considering that the periphery of the tumor is supposed to be the most important area with the highest biological relevance representing the invasive front, further analyses concentrated on this localization [35,36]. The expression of the different MMPs and TIMP-1 was analyzed by comparing the different groups at the same time-point and same group over time (Figure 9). 

The MMP-2-positive area within the tumor periphery was at 54 dpt significantly larger in DH82-CDVai xenografts compared to untreated controls (*p* < 0.0001) and DH82-UV-CDVai tumors (*p* = 0.0005). In addition, a significantly larger peripheral area expressing MMP-2 was noted at 63 dpt in DH82-UV-CDVai neoplasms compared to untreated controls (*p* = 0.0017) and DH82-medium xenografts (*p* = 0.0038). All other comparisons between different groups at the same time point lacked significant differences. A comparison of the MMP-2 expression over time revealed a significantly smaller peripheral immunopositive area in non-treated controls at 54 dpt (*p* < 0.0001) and 63 dpt (*p* < 0.0001) compared to 44 dpt. Similarly, DH82-medium xenografts comprised a smaller-sized MMP-2-positive area at 54 dpt (*p* = 0.0036) and 63 dpt (*p* < 0.0001) within the tumor periphery compared to 44 dpt. An opposite development was noted in DH82-UV-CDVai xenografts, which exhibited a larger peripheral MMP-2 immunoreactive area at 63 dpt compared to 54 dpt (*p* = 0.0059). Besides the aforementioned, no significant differences were present over time within the different groups (Figure 9A).

The peripheral MMP-9 immunopositive area was significantly larger in DH82-CDVai xenografts than in all other groups at 44 dpt (*p* < 0.0001). A similar result was obtained at 63 dpt. At this time point DH82-CDVai neoplasms exhibited a larger peripheral MMP-9 immunopositive area than DH82-UV-CDVai tumors (*p* = 0.0033) or DH82-medium xenografts (*p* < 0.0001). Furthermore, a significantly larger peripheral MMP-9 area was observed in DH82-medium tumors at 63 dpt compared to untreated control neoplasms (*p* = 0.0002). All other comparisons lacked statistically significant differences. Interestingly, the peripheral MMP-9 expression in DH82-CDVai xenografts decreased significantly over time (*p* values ranging from <0.0001 to 0.0012). With the exception of DH82-medium xenografts, which exhibited an increase in the peripheral MMP-9 immunopositive area from 44 dpt to 63 dpt (*p* = 0.0428), all other groups lacked statistically significant changes over time (Figure 9B).

The peripheral MMP-14 immunopositive area was significantly smaller in DH82-CDVai xenografts compared to all control groups at 63 dpt (*p* < 0.0001). Additionally, untreated control neoplasms revealed a smaller MMP-14 immunopositive area at 44 dpt compared to DH82-UV-CDVai tumors (*p* < 0.0001). No other significant changes were observed between the other groups independent of the time point. Interestingly, DH82-CDVai xenografts revealed a decrease in the peripheral MMP-14 immunopositive area from 44 dpt to 63 dpt (*p* = 0.0001). In contrast, DH82-UV-CDVai and DH82-medium tumors developed a reduction of the peripheral MMP-14 immunopositive area from 44 dpt to 54 dpt (*p* = 0.0004 and *p* < 0.0001). This decrease was followed by significant enlargement of the peripheral MMP-14 immunopositive area from 54 dpt to 63 dpt in both groups (*p* < 0.0001). All other groups lacked statistically significant changes over time (Figure 9C).

At 44 dpt, the peripheral TIMP-1 expressing area in DH82-UV-CDVai tumors was significantly smaller than in all other groups (*p* values ranging from <0.0001 to 0.0014). A contrary result was obtained at 54 dpt. At this time point, DH82-UV-CDVai neoplasms exhibited significantly larger peripheral TIMP-1 immunopositive areas than xenografts of all other groups (*p* < 0.0001). At the last time point investigated (63 dpt), DH82-CDVai tumors revealed a significantly smaller peripheral TIMP-1 immunopositive area than DH82-medium xenografts (*p* < 0.0001). Furthermore, DH82-medium neoplasms displayed a smaller peripheral TIMP-1 immunopositive area than DH82-UV-CDVai (*p* < 0.0001) xenografts or untreated controls (*p* < 0.0001). Interestingly, DH82-CDVai, DH82-medium tumors and untreated controls showed a significant smaller peripheral TIMP-1 immunopositive area at 54 dpt (*p* < 0.0001) and 63 dpt (*p* < 0.0001) compared to 44 dpt. In DH82-UV-CDVai xenografts, an increase in the peripheral TIMP-1 immunopositive area was observed from 44 dpt to 54 dpt (*p* = 0.0124), followed by a decrease from 54 dpt to 63 dpt (*p* < 0.0001). This resulted in a significantly smaller peripheral TIMP-1 immunopositive area at 63 dpt compared to 44 dpt (*p* =0.073; Figure 9D). All data are reported in Supplementary Table S2.

Summarized, DH82-CDVai xenotransplants possessed a higher number of tumor-associated macrophages. Furthermore, an altered MMP and TIMP expression was present at the invasive front of the xenografts.

### 2.8. Acute CDV-Infection Influences Microvascular Density in DH82 Xenografts

**Figure 10:**
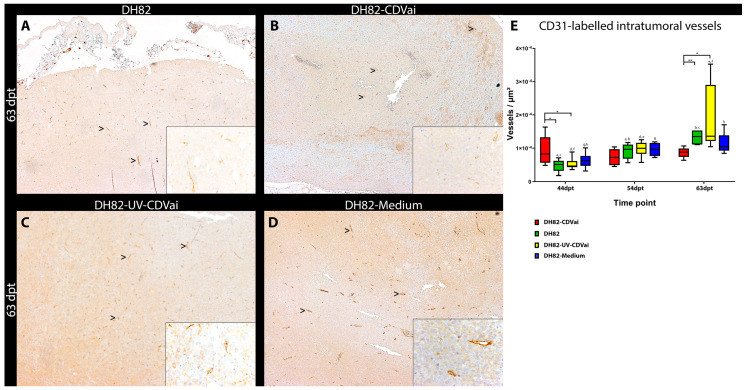
CD31 immunolabeling in murine subcutaneous DH82 cell xenografts for assessment of microvessel density. All CD31-expressing structures with a definable lumen were counted as vessels (arrowheads). (**A**) displays non-infected (DH82), (**B**) intratumorally canine distemper virus (CDV)-infected (DH82-CDVai), (**C**) intratumorally UV-inactivated CDV-injected (DH82-UV-CDVai) and (**D**) intratumorally medium-injected (DH82-medium) neoplasms at 63 days post-transplantation (dpt). (**E**) shows a graphical overview of the microvessel density within the different groups. All graphs represent Box and whisker plots with statistically significant differences between different groups at the same time point (* *p* < 0.05 and ** *p* ≤ 0.01) and between different time points within the same group (a–h; *p* < 0.05). Scale bar = 100 µm and scale bar—insert = 20 µm.

The microvessel density in whole tumor sections was determined to elucidate whether it is influenced by CDV infection of tumor cells. At 44 dpt, DH82-CDVai neoplasms exhibited a significantly higher number of vessels per area compared to DH82-UV-CDVai (*p* = 0.0303) and untreated controls (*p* = 0.0453). At 63 dpt, a significantly lower microvessel density was present in DH82-CDVai xenografts compared to DH82-UV-CDVai (*p* = 0.0137) and untreated controls (*p* = 0.0080). All other groups lacked significant differences in the microvessel density at the respective time point. The microvessel density significantly increased over time in all control groups (*p* values ranging from 0.0050 to 0.0202), with the exception of DH82-medium xenografts lacking a further increase in microvessel density from 54 dpt to 63 dpt. A contrary finding was present in DH82-CDVai tumors, which displayed a stable number of vessels per area as reflected by lacking statistically significant differences over time (Figure 10). All data are reported in Supplementary Table S1.

Summarized, these findings revealed that an intratumoral CDV-Ond infection inhibits an increase of the microvessel density within infected xenografts over time.

## 3. Discussion

The use of oncolytic virotherapy represents a promising approach in the treatment of various neoplasms, as demonstrated in xenotransplantation studies and clinical trials [28,37,38]. On the other hand, in vitro investigations and xenotransplantation studies, especially in immunocompromised animals, are not reflecting the spontaneous disease in the natural host in all aspects. In viral oncolysis, it has to be taken into account that many diseased animals will possess antiviral antibodies when using a core vaccine component like CDV. Interestingly, it has been demonstrated in diseased humans, treated intratumorally with measles virus, that pre-existing anti-measles antibodies did not affect therapeutic success [39]. Furthermore, intratumoral virus distribution might be affected by larger-sized tumors within dogs, necessitating larger virus loads or simultaneous injections into different localizations within the tumor, which might be impossible in animals with disseminated disease involving many internal organs.

However, detailed studies regarding new therapies and the complex mechanisms of action on a cellular and molecular level are difficult to investigate in unpredictable, spontaneous tumors. To overcome these limitations, translational mouse models with xenotransplantation of tumor cells are essential to standardize environmental conditions, treatment schemes and chronological events in pathogenetic mechanisms.

Previous studies demonstrated that persistently infected DH82 cell xenografts displayed a complete tumor regression after the initial development of solid neoplasms [34]. To overcome the limitations originating from the xenotransplantation of persistently CDV-infected DH82 cells, the present study concentrates on the clinically more relevant model of intratumoral infection of established non-infected xenografts. Furthermore, the present investigation focused on the modulation of the tumor microenvironment, which might enhance the therapeutic efficacy of oncolytic CDV (strain Onderstepoort) in canine histiocytic sarcoma cell (DH82 cell) xenografts.

Established DH82 cell xenografts showed a significantly slower growth rate after a tenfold intratumoral CDV infection than controls injected either with UV-inactivated CDV or medium or left untreated (non-infected DH82 xenografts). Interestingly, this was associated with large areas of necrosis within CDV-infected tumors, which were only detectable in limited amounts in control groups, while apoptosis seems to play a minor role in all groups. In contrast to the present findings, cell death via apoptosis was suspected in CCT cells, another canine histiocytic sarcoma cell line, after infection with CDV [40]. This is in line with studies demonstrating the effect of an intratumoral treatment of xenotransplanted human lung and colorectal carcinoma cells with attenuated measles virus strains closely related to CDV. These infections resulted in a high apoptotic rate of neoplastic cells with consecutive prolonged survival of treated animals [41,42]. The necrosis observed in the present investigations might result from a reduced microvessel density leading to hypoxic cell death.

The concept of reduced angiogenesis as a viral oncolysis mechanism has been described before for various virus families, including Poxviridae and Rhabdoviridae [26,43,44]. Similarly, persistently CDV-infected DH82 xenografts displaying a complete spontaneous regression showed a lower microvessel density than non-infected controls [34]. The less pronounced effect in the present study lacking complete regression after intratumoral CDV infection might result from an inhibition of virus spread within the neoplastic tissue (<0.5% of tumor area infected) compared to typically high numbers of infected cells (>85%) in persistently infected cultures [34,45]. The mechanism of reduced angiogenesis might be addressed to an impairment of the hypoxia-inducible factor-1 α (HIF-1α) downstream pathway that resulted in a decreased vascular endothelial growth factor-B (VEGF-B) expression in persistently CDV-infected DH82 cells in vitro [46].

An important factor interfering with virus spread and angiogenesis within neoplasms is represented by the tumor microenvironment [47], with mediators modulated by oncolytic viruses gaining increasing attendance. These mediators include matrix metalloproteinases (MMPs) and their inhibitors (tissue inhibitors of metalloproteinases, TIMPs) which are linked to extracellular matrix turnover and angiogenesis [48]. Furthermore, several studies demonstrated modulation of MMPs and their inhibitors in vitro and in spontaneously occurring canine CDV infections [31,49,50,51,52]. Similarly, the present study demonstrates a CDV-associated modulation of the MMP-2, -9, -14 and TIMP-1 expression within subcutaneous xenografts. The most interesting finding in this context is the continuously decreasing expression of MMP-9 and TIMP-1 at the invasive front of the tumor periphery in CDV-infected xenografts over time, which accompanied the decelerated growth of infected neoplasms. In the literature, a high expression of MMPs, especially MMP-9, has been linked to poor prognosis in several tumor types often associated with an increased risk of invasion and metastasis [53,54]. Therefore, the virus-induced reduction of the MMP-9 expression might result in a decreased invasive potential of canine histiocytic sarcoma xenografts. Furthermore, MMP-9 is known to favor tumor angiogenesis [55], and its reduced expression might, therefore, represent one factor contributing to the low microvessel density observed in CDV-infected DH82 xenografts. The relevance of a reduced TIMP-1 expression on tumor progression is discussed controversially within the literature [56]. Therefore, the exact role of this protein on reduced angiogenesis and tumor cell growth could not be elucidated within the present study. However, an altered MMP-9/TIMP-1 ratio is assumed to influence tumor progression [56].

MMPs and TIMP-1 are often secreted directly by tumor cells [31,57]. However, other components of the tumor microenvironment, such as stromal cells and tumor-associated macrophages, are also important sources of these proteins [48]. It has been shown previously that spontaneous canine histiocytic sarcomas exhibited an expression of MMPs and TIMP-1, mainly at the invasive front of the neoplasms and within tumor-associated macrophages [32]. Similar findings were observed for non-infected subcutaneously xenotransplanted canine histiocytic sarcoma cells (DH82 cells) (32). Within the present study, tumor-associated macrophages were present in moderate numbers within intratumorally CDV-infected neoplasms, while there was only a scant infiltration in all control groups until 54 dpt. The lacking significant difference at 63 dpt might be attributed to the limited virus distribution after the last intratumoral virus injection. However, tumor-associated macrophages might represent an important part of an anti-tumoral immune response. Another important factor attracting macrophage infiltration might be represented by the amount of necrosis inducing a resorptive inflammation. Nevertheless, tumor-associated macrophages might have either an anti-tumor M1 or pro-tumoral M2 phenotype within the tumor microenvironment rendering a precise prediction about their function difficult [58,59,60]. Therefore, tumor-associated macrophages’ polarization represents an attractive emerging target for therapeutic intervention with oncolytic viruses, which needs to be detailed in further studies.

Summarized, the present study demonstrates that an intratumoral CDV-infection of canine histiocytic sarcoma xenografts led to reduced tumor growth. In this context, inhibition of angiogenesis in combination with an enhancement of the innate immune response seems to play an important role in modulating the expression of matrix modifying enzymes as one factor. However, the exact interplay between angiogenesis and innate immune response as represented by tumor-associated macrophages needs to be detailed in further studies.

## 4. Materials and Methods

### 4.1. Cell Culture

DH82 cells, a permanent canine histiocytic sarcoma cell line, were obtained from the European Collection of Authenticated Cell Cultures (ECACC No. 94062922). Cultivation of DH82 cells for grafting was carried out in standard conditions as previously described [33,34,45,46]. To ensure maximum viability for grafting, cells were harvested at 70% to 80% confluence of the monolayer. Cells were obtained by scraping, rinsing in a cell culture medium without supplementation of fetal bovine serum (FBS, PAA, Cölbe, Germany) and nonessential amino acids (NEAA, Sigma-Aldrich, Taufkirchen, Germany) and centrifugation.

### 4.2. Establishment of Murine Xenografts

A total number of 96 female severe combined immunodeficiency (SCID) mice (CB17/Icr-Prkdcscid/lcrlcoCrl) were obtained from a commercial breeder (Charles River Wiga, Sulzfeld, Germany) at 4 weeks of age. Mice were kept in groups of 6 in individually ventilated cage systems (Tecniplast, Hohenpeißenberg, Germany) and had an adaptation period of 2 weeks. Mice were subcutaneously xenotransplanted with DH82 cells as previously described [32,34]. Briefly, 3.0 ∗ 10^6^ DH82 cells in 100 µL FBS- and NEAA-free medium were injected once into the subcutis of each animal’s left flank using single-use pen needles (Omnican^®^ F, B. Braun, Melsungen, Germany). Subsequently, tumor development was monitored every second to the third day by measuring tumor width and length with an analog vernier precision caliper (C. Schulz measuring instruments, Kloster Lehnin, Germany). Tumor volume was calculated as ((shortest diameter^2^ × longest diameter)/2) [61]. Furthermore, total body weight was determined.

### 4.3. Intratumoral Treatment of Xenotransplanted Histiocytic Sarcomas

Starting 35 days after xenotransplantation when tumors exhibited a diameter of ~0.5 cm (≙0.06 cm^3^), 3 groups of mice (*n* = 6 per group and time point) were injected ten times every second day with (1) 100 µL intratumoral injections of CDV, strain Onderstepoort (CDV-Ond; TCID_50_ = 10^4.5^/100 µL; DH82-CDVai xenografts) or (2) UV-inactivated CDV-Ond (DH82-UV-CDVai xenografts) or (3) medium (DH82-medium xenografts) in the same amount and following the same treatment scheme as controls. Non-treated DH82 xenografts (*n* = 6 per time point) served as additional controls (non-infected DH82 xenografts). Tumor growth was monitored as described above. In addition, mice were controlled for changes in body weight and signs of adverse reactions. Necropsies were performed, and tumors were sampled on days 44, 54, 63 and 77 after transplantation (dpt). Animals were preliminary sacrificed when tumors exceeded a volume of 1.7 cm^3^ (equals a diameter of approx. 1.5 cm).

### 4.4. Histology and Immunohistochemistry

Tumor tissue was fixed for 24 h in 10% neutral-buffered formalin and subsequently routinely processed and embedded into paraffin blocks. Serial sections (2–4 μm) were either stained with hematoxylin–eosin (HE) for standard histology or used for immunohistochemical analysis.

Immunohistochemistry (IHC) was performed by applying the avidin-biotin-peroxidase complex (ABC, Vector Labs, Burlingame, CA, USA) procedure as previously described [34,49,62,63]. Details about the procedure and antibodies used are given in Table 1. DH82 cells of xenotransplants were visualized using a CD44 antibody, which does not cross-react with murine tissue [64]. An antibody directed against the nucleoprotein of CDV was used to analyze the intratumoral virus amount and distribution in DH82-CDVai and DH82-UV-CDVai xenografts. For marking apoptotic tumor cells, an antibody directed against cleaved caspase 3 was used (66). Microvessel density within the tumor microenvironment was analyzed by using an antibody directed against CD31 [65] and counting all structures with a discernable lumen. Tumor-associated macrophages were marked using an antibody directed against Mac3 (CD107b/LAMP2), which shows no cross-reaction with canine histiocytic sarcoma cells [32,66,67]. Furthermore, the expression of MMPs and TIMPs was evaluated by using antibodies directed against MMP-2, MMP-9, MMP-14 and TIMP-1 [36]. The cross-reactivity of the aforementioned MMP-2, -9, -14 and TIMP-1 antibodies has been previously verified using western blot [31,32].

For negative controls, specific primary antibodies were replaced by ascitic fluid from non-immunized BALB/cJ mice (for CDV and MMP-2), an anti-rat IgG1 isotype antibody (for Mac3), rat serum from non-immunized rats (for CD44) and serum from non-immunized rabbits (for cleaved caspase-3, MMP-9, MMP-14, TIMP-1, and CD31). The dilution of negative controls was chosen according to the protein concentration of replaced primary antibodies.

### 4.5. Histological and Immunohistochemical Analysis, Including Morphometric Analysis

HE-stained tumor slides were morphometrically analyzed to determine total tumor area and to quantify necrosis. Therefore, HE stained slides containing a cross-section of the complete tumor diameter were digitized (Mirax Scanner, Zeiss, Jena, Germany), and morphometry was performed using the Aperio ImageScope software (version 12, Leica Aperio Technologies, Wetzlar, Germany). The percentage of necrotic tumor areas was determined by manually contouring the boundaries of each neoplasm to adjacent subcutaneous tissue, as were the areas of necrosis within the tumor. Calculation of corresponding areas was stated in µm^2^. Afterwards, the percentage of necrotic tumor area was calculated by dividing necrotic area by total tumor area and multiplying the result by 100.

Similarly, immunohistochemically stained slides using antibodies directed against CDV, Mac3 (CD107b/LAMP2) and cleaved caspase-3 were digitized. Morphometry was performed using the Aperio ImageScope software (version 12, Leica Aperio Technologies, Wetzlar, Germany).

The area of chromogen expression was determined within the total area of the grafted tumor tissue (“region of interest”) and stated as positive pixels/µm^2^ total tumor area. Thresholds of positive pixel count v9 algorithm for each staining were manually adjusted according to staining intensity.

For MMPs and TIMP-1, pictures from each xenotransplant were taken at a magnification of ×400 in 10 randomly selected areas of tumor center and periphery, respectively, using the Cell-D imaging software (Olympus Soft Imaging Solutions, Münster, Germany). Obtained pictures were analyzed using the positive pixel count algorithm of the Aperio ImageScope viewer (Version 12, Aperio Technologies). Only areas containing neoplastic tissue were analyzed. Tumor-free regions, necrotic areas and artifacts (e.g., tissue folding) were excluded from further analysis. For the positive pixel count algorithm, hue value of 0.1 and hue width of 0.5, color saturation threshold of 0.04 were used, and any intensity of staining was considered positive. The number of positive pixels was divided by the total number of pixels (negative and positive) in the analyzed area and multiplied by 100 to receive the percentage of positive pixels. Values from each section were averaged.

Microvessel density analysis within the xenografts was performed by manually counting the number of CD31-positive structures containing a definite lumen as described before [34]. Twenty-five (25) visual fields were analyzed at a 400× magnification using light microscopy (Axiostar Plus, Carl Zeiss Microscopy GmbH). The density of vascularization was indicated as a number of positive structures exhibiting a lumen per μm^2^ tumor area.

### 4.6. Statistical Analysis

Statistical analysis of tumor volume development, histological and immunohistological evaluation was calculated using the Wilcoxon rank-sum test with the significance level *p* < 0.05. Analyses were performed with the Statistical Analysis System, Version 9.3 (SAS Institute Inc., Heidelberg, Germany).

Analysis of immunohistochemical data for MMPs, TIMP-1, including the difference in MMP and TIMP-1 expression between different groups and between central and peripheral areas of the xenotransplanted histiocytic sarcomas was performed using the Wilcoxon signed-rank test with a Tukey–Kramer post hoc adjustment.

Graphical illustrations were designed with GraphPad Prism, Version 8.0.1 (GraphPad Software Inc., San Diego, CA, USA).

## Figures and Tables

**Table 1:** Summary of used antibodies in immunohistochemical methods for the detection of canine distemper virus, tumor cells, apoptosis, matrix metalloproteinases and their inhibitors, microvessel density and tumor-associated macrophages in xenotransplanted canine histiocytic sarcomas.

Antibody	Name, Clonality, Source	Demasking Procedure	Dilution of Antibodies	Secondary Antibody	Positive Control
**CD44**	Clone 2D10; monoclonal rat; E. Kremmer, München, Germany	Microwave/CB 20 min	1:200	RaR-b	DH82 cell pellet
**CDV-NP**	Clone D110; monoclonal mouse; A. Zurbriggen, Bern, Switzerland	Microwave/CB 20 min	1:100	GaM-b	persistently CDV-Ond-infected DH82 cell pellet
**Cleaved** **Caspase 3** **(Asp175)**	Polyclonal rabbit; Cell Signaling Technology Inc., Danvers, MA, USA	Microwave/CB 20 min	1:200	GaR-b	SCID mouse, lymphoid tissue
**Mac3 (CD107b/LAMP2)**	Monoclonal rat; clone: M3/84; AbD Serotec, Oxford, UK	Microwave/CB 20 min	1:200	n.a.	SCID mouse, lymphoid tissue
**CD31**	Polyclonal rabbit; Acris antibodies, Hiddenhausen, Germany	Microwave/CB 20 min	1:100	GaR-b	Canine granulation tissue
**MMP-2**	MAB13405; monoclonal mouse; Millipore, Burlington, MA, USA	None	1:400	GaM-b	DH82 cell pellet
**MMP-9**	RM105-MMP9; polyclonal rabbit; Triple Point Biologics, Forest Grove, OR, USA	None	1:500	GaR-b	DH82 cell pellet
**MMP-14**	RP1-MMP14; polyclonal rabbit; Triple Point Biologics, Forest Grove, OR, USA	None	1:200	GaR-b	DH82 cell pellet
**TIMP-1**	RP3-TIMP1; polyclonal rabbit; Triple Point Biologics, Forest Grove, OR, USA	None	1:1000	GaR-b	Canine stillborn puppy, bone marrow

CDV-N = canine distemper virus nucleoprotein; GaM-b = goat-anti-mouse antibody biotinylated; GaR-b = goat-anti-rat antibody biotinylated; n.a. = not applicable; RaR-b = rabbit-anti-rat antibody biotinylated; MMP = matrix metalloproteinase; TIMP = tissue inhibitor of matrix metalloproteinase; CB = citrate buffer.

## Data Availability

All relevant data are included in the manuscript, supplementary material or can be obtained from the authors on reasonable request.

## Supplementary Materials

The following are available online at https://www.mdpi.com/article/10.3390/ijms22073578/s1, Table S1: Summary of intratumoral CDV amount, necrosis, apoptosis, microvessel density and tumor-associated macrophages in acutely CDV-infected DH82 xenografts and corresponding controls, Table S2: Immunohistochemical expression of MMP-2, MMP-9, MMP-14 and TIMP-1 within acutely CDV-infected DH82 xenotransplants and control groups.
