# Supplementary material for: Intratumoral Canine Distemper Virus Infection Inhibits Tumor Growth by Modulation of the Tumor Microenvironment in a Murine Xenograft Model of Canine Histiocytic Sarcoma
Source: Int J Mol Sci. 2021 Mar 30;22(7):3578. doi: 10.3390/ijms22073578 (PMC8037597; doi:10.3390/ijms22073578)
Supplement: Supplementary file 1 [file ijms-22-03578-s001.pdf]

International Journal of *Molecular Sciences*

MDPI

Article

Intratumoral Canine Distemper Virus Infection Inhibits Tumor Growth by Modulation of the Tumor Microenvironment in a Murine Xenograft Model of Canine Histiocytic Sarcoma

Federico Armando [1,†], Adnan Fayyad [1,2,†], Stefanie Arms [1], Yvonne Barthel [1], Dirk Schaudien [3], Karl Rohn [4], Matteo Gambini [1,5], Mara Sophie Lombardo [1], Andreas Beineke [1], Wolfgang Baumgärtner [1,*] and Christina Puff [1]

1 Department of Pathology, University of Veterinary Medicine Hannover, Bünteweg 17, 30559 Hannover, Germany; federico.armando@tiho-hannover.de (F.A.); adnanf@najah.edu (A.F.); stefanie.arms@boehringer-ingelheim.com (S.A.); ybbarthel@aol.de (Y.B.); matteo.gambini@unimi.it (M.G.); mara.sophie.lombardo@tiho-hannover.de or mara.lombardo@tiho-hannover.de (M.S.L.); andreas.beineke@tiho-hannover.de (A.B.); christina.puff@tiho-hannover.de (C.P.)
2 Department of Veterinary Medicine, An-Najah National University, Nablus 9720061, Palestine
3 Fraunhofer Institute for Toxicology and Experimental Medicine, Nikolai-Fuchs-Straße 1, 30625 Hannover, Germany; dirk.schaudien@item.fraunhofer.de
4 Institute for Biometry, Epidemiology and Information Processing, University of Veterinary Medicine Hannover, Bünteweg 2, 30559 Hannover, Germany; karl.rohn@tiho-hannover.de
5 Dipartimento di Medicina Veterinaria (DIMEVET), Università degli Studi di Milano, Via dell'Università 6, 26900 Lodi, Italy
* Correspondence: wolfgang.baumgaertner@tiho-hannover.de; Tel.: +49-511-953-8620
† These authors contributed equally to this work and are listed in alphabetical order.

Citation: Armando, F.; Fayyad, A.; Arms, S.; Barthel, Y.; Schaudien, D.; Rohn, K.; Gambini, M.; Lombardo, M.S.; Beineke, A.; Baumgärtner, W.; et al. Intratumoral Canine Distemper Virus Infection Inhibits Tumor Growth by Modulation of the Tumor Microenvironment in a Murine Xenograft Model of Canine Histiocytic Sarcoma. *Int. J. Mol. Sci.* **2021**, *22*, 3578. https://doi.org/10.3390/ijms22073578

Academic Editor: Laura Menotti

Received: 5 March 2021
Accepted: 26 March 2021
Published: 30 March 2021

Publisher's Note: MDPI stays neutral with regard to jurisdictional claims in published maps and institutional affiliations.

Copyright: © 2021 by the authors. Licensee MDPI, Basel, Switzerland. This article is an open access article distributed under the terms and conditions of the Creative Commons Attribution (CC BY) license (http://creativecommons.org/licenses/by/4.0/).

Supplementary material:

This file includes:

Tables: 2

Supplementary table S1: Summary of intratumoral CDV amount, necrosis, apoptosis, microvessel density and tumor-associated macrophages in acutely CDV-infected DH82 xenografts and corresponding controls

Marker	Group	44 dpt	54 dpt	63 dpt
**CDV** (% of tumor area)	Non-infected DH82	n.e.	n.e.	n.e.
	DH82-CDVai	0.126 * (0.053; 0.402)	0.227 * (0.066; 0.304)	0.121 * (0.005; 0.347)
	DH82-UV-CDVai	0.013 * (0.002; 0.017)	0.003 * (0.003; 0.012)	0.003 * (0.002; 0.003)
	DH82-Medium	n.e.	n.e.	n.e.
**Necrosis** (% of tumor area)	Non-infected DH82	0.535 * (0.000; 1.260)	0.450 * (0.410; 2.380)	0.490 *† (0.040; 0.930)
	DH82-CDVai	18.710 $^{*\dagger\#}$ (3.090; 43.700)	15.950 $^{*\dagger\#}$ (2.910; 31.410)	12.910 $^{*\#}$ (1.020; 16.150)
	DH82-UV-CDVai	1.365 † (0.000; 17.010)	1.540 † (0.460; 7.470)	1.070 $^{\#}$ (0.050; 74.000)
	DH82-Medium	0.555 $^{\#}$ (0.270; 5.680)	2.115 $^{\#}$ (0.120; 23.080)	1.580 † (0.910; 5.210)
**Cleaved caspase 3** (% of tumor area)	Non-infected DH82	0.209 ^{*a} (0.096; 0.413)	0.038 ^{*a} (0.017; 0.154)	0.115 (0.019; 0.898)
	DH82-CDVai	1.273 $^{*\dagger\#ab}$ (0.301; 4.010)	0.340 $^{*\dagger\#ac}$ (0.252; 0.535)	0.241 ^{bc} (0.060; 0.263)
	DH82-UV-CDVai	0.264 † (0.011; 0.612)	0.085 $^{\dagger a}$ (0.020; 0.338)	0.347 ^{a} (0.085; 1.779)
	DH82-Medium	0.088 $^{\#}$ (0.012; 0.309)	0.162 $^{\#}$ (0.034; 0.359)	0.163 (0.092; 0.230)

Supplementary table S1 cont.

Marker	Group	44 dpt	54 dpt	63 dpt
**CD31** (positive structures/μm^2)	Non-infected DH82	5.10×10^{-5} $^{*\,a\,b}$ (1.80×10^{-5}; 7.10×10^{-5})	9.66×10^{-5} $^{a\,c}$ (5.65×10^{-5}; 11.70×10^{-5})	13.49×10^{-5} $^{*\,b\,c}$ (11.08×10^{-5}; 15.36×10^{-5})
	DH82-CDVai	8.23×10^{-5} $^{*\,\dagger}$ (4.84×10^{-5}; 16.35×10^{-5})	7.23×10^{-5} (4.51×10^{-5}; 10.40×10^{-5})	8.83×10^{-5} $^{*\,\dagger}$ (6.36×10^{-5}; 10.68×10^{-5})
	DH82-UV-CDVai	4.64×10^{-5} $^{\dagger\,a\,b}$ (3.58×10^{-5}; 8.86×10^{-5})	9.98×10^{-5} $^{a\,c}$ (5.72×10^{-5}; 12.55×10^{-5})	13.60×10^{-5} $^{\dagger\,\#\,b\,c}$ (10.45×10^{-5}; 35.27×10^{-5})
	DH82-Medium	6.25×10^{-5} $^{a\,b}$ (3.15×10^{-5}; 10.10×10^{-5})	9.61×10^{-5} $^{a\,c}$ (7.20×10^{-5}; 11.90×10^{-5})	10.49×10^{-5} $^{\#\,b\,c}$ (8.45×10^{-5}; 17.05×10^{-5})
**Mac3 (CD107b/LAMP2)** (% of tumor area)	Non-infected DH82	0.004 $^{*\,\dagger\,a}$ (0.002; 0.068)	0.013 $^{*\,\dagger}$ (0.008; 0.077)	0.050 ^{a} (0.008; 0.269)
	DH82-CDVai	0.387 $^{*\,\#\,\S}$ (0.078; 1.433)	0.379 $^{*\,\#\,\S}$ (0.310; 1.360)	0.203 (0.035; 0.597)
	DH82-UV-CDVai	0.068 $^{\dagger\,\#}$ (0.011; 0.180)	0.048 $^{\dagger\,\#}$ (0.036; 0.386)	0.114 (0.070; 1.878)
	DH82-Medium	0.023 $^{\S\,a\,b}$ (0.001; 0.081)	0.190 $^{\S\,a}$ (0.006; 0.302)	0.100 ^{b} (0.047; 0.512)

The values are displayed as median (minimum; maximum).

CDV: Canine distemper virus; CD31 = evaluation of microvessel density; Cleaved caspase 3 = detection of apoptotic cells; DH82-CDVai = DH82 cell xenotransplants with intratumoral infection with CDV-Ond; DH82-UV-CDVai = DH82 cell xenotransplants with intratumoral injection of UV-inactivated CDV; Mac3 (CD107b/LAMP2) = detection of tumor infiltrating murine macrophages; n.e. = not evaluated; Non-infected DH82 = non-infected DH82 cell xenotransplants;

($^{*\,\dagger\,\#\,\S}$) represent statistically significant differences ($p < 0.05$) between different groups at the same time point;

($^{a\,b\,c}$) represent statistically significant differences ($p < 0.05$) between different time points for the same group at the same location.

Supplementary table S2: Immunohistochemical expression of MMP-2, MMP-9, MMP-14 and TIMP-1 within acutely CDV-infected DH82 xenotransplants and control groups

	**Time point**	**44 dpt**		**54 dpt**		**63 dpt**	
**Marker**	**Group**	**C**	**P**	**C**	**P**	**C**	**P**
**MMP-2** (% of tumor area)	Non-infected DH82	19.16 (1.20; 86.82)	82.38 [a b] (27.62; 96.06)	18.00 (1.46; 36.05)	44.38 [* a] (21.39; 69.51)	20.41 (1.37; 47.79)	45.53 [* b] (18.79; 75.59)
	DH82-CDVai	23.19 (1.10; 73.01)	71.05 (38.95; 91.89)	22.94 (1.96; 71.30)	68.06 [* †] (47.19; 82.94)	50.40 (7.85; 77.25)	72.58 (17.77; 84.65)
	DH82-UV-CDVai	4.83 (1.01; 82.17)	56.75 (21.19; 91.11)	14.03 (1.12; 27.80)	50.35 [† a] (25.46; 80.61)	25.58 (2.52; 71.59)	65.81 [* † a] (35.99; 82.61)
	DH82-Medium	21.30 (1.15; 40.78)	71.92 [a b] (38.33; 92.08)	13.98 (1.12; 51.98)	54.71 [a] (20.74; 91.91)	28.04 (2.94; 53.10)	51.03 [† b] (12.89; 81.58)
**MMP-9** (% of tumor area)	Non-infected DH82	11.17 (1.46; 72.49)	61.52 [*] (4.84; 89.72)	31.07 (1.27; 67.56)	61.22 (3.96; 94.85)	32.78 (1.37; 76.02)	53.49 [*] (1.02; 86.57)
	DH82-CDVai	38.98 (4.41; 88.71)	84.57 [* † # a b] (60.28; 94.68)	31.95 (5.18; 81.80)	65.19 [a c] (14.48; 96.57)	12.12 (2.11; 43.83)	42.36 [† # b c] (2.23; 76.83)
	DH82-UV-CDVai	6.48 (1.06; 34.47)	45.15 [†] (15.49; 79.95)	17.68 (1.13; 70.25)	55.98 (12.18; 89.90)	34.74 (7.52; 70.42)	59.94 [†] (6.32; 92.63)
	DH82-Medium	3.84 (0.00; 12.47)	60.87 [# a] (1.24; 93.64)	16.20 (1.11; 60.16)	56.05 (22.20; 80.13)	50.69 (1.01; 78.53)	68.26 [* # a] (14.21; 99.50)

Supplementary table S2 cont.

	**Time point**	**44 dpt**		**54 dpt**		**63 dpt**	
**Marker**	**Group**	**C**	**P**	**C**	**P**	**C**	**P**
**MMP-14** (% of tumor area)	Non-infected DH82	37.82 (2.34; 98.46)	78.21 * (10.26; 98.84)	30.63 (0.13; 96.79)	78.61 (50.25; 96.02)	66.84 (1.81; 86.69)	89.84 * (19.14; 98.26)
	DH82-CDVai	17.19 (1.99; 93.84)	84.73 a (60.38; 98.78)	30.36 (1.47; 69.49)	77.88 (43.91; 92.79)	66.90 (7.18; 96.28)	62.89 * † # a (23.92; 93.59)
	DH82-UV-CDVai	25.87 (3.77; 89.80)	95.14 * a (57.61; 99.77)	30.27 (1.10; 97.77)	78.43 a b (28.81; 95.57)	98.48 (80.25; 99.78)	98.54 † b (84.09; 99.98)
	DH82-Medium	17.83 (0.71; 96.46)	94.81 a (19.54; 99.93)	36.52 (1.25; 91.73)	65.44 a b (17.06; 99.11)	67.54 (7.34; 96.91)	91.21 # b (25.69; 99.57)
**TIMP-1** (% of tumor area)	Non-infected DH82	13.55 (1.03; 91.14)	94.62 * a b (43.92; 99.72)	21.21 (1.85; 82.48)	68.97 * a (14.25; 95.28)	29.23 (7.50; 75.92)	53.80 * b (16.68; 95.25)
	DH82-CDVai	7.53 (1.43; 89.66)	94.21 † a b (78.90; 99.54)	13.65 (1.38; 72.09)	56.10 † a (9.17; 100.00)	12.68 (1.76; 46.06)	46.49 † b (17.49; 80.47)
	DH82-UV-CDVai	8.32 (0.56; 69.75)	81.76 * † # a b (4.33; 98.07)	34.04 (0.42; 88.59)	91.43 * † # a c (38.00; 99.84)	27.04 (2.54; 67.46)	58.69 # b c (10.86; 85.73)
	DH82-Medium	5.13 (1.02; 51.43)	90.09 # a b (60.16; 98.19)	8.36 (1.09; 66.90)	62.17 # a (14.50; 89.08)	18.38 (1.81; 64.48)	82.10 * † # b (34.22; 93.96)

Values are depicted as median (minimum; maximum) of % positively stained tumor area. Please note that group wise comparisons and evaluations over time focused on peripheral tumor areas only as they represent the invasive front of the tumor.

C = tumor center; CDV-Ond = canine distemper virus (strain Onderstepoort); DH82-CDVai = DH82 cell xenotransplants with intratumoral infection with CDV-Ond; DH82-Medium = DH82 cell xenotransplants with intratumoral injection of Medium; DH82-UV-CDVai = DH82 cell xenotransplants with intratumoral injection of UV-inactivated CDV; dpt = days after transplantation; MMP = matrix metalloproteinase; non-infected DH82 = non-infected DH82 cell xenotransplants; P = tumor periphery; TIMP = tissue inhibitor of matrix metalloproteinase; P = tumor periphery;

Gray shading represents a statistically significant difference ($p < 0.05$) between different localizations (center vs. periphery) for the same group at the same time point;

(* † #) represent statistically significant differences ($p < 0.05$) between peripheral areas of different groups at the same time point;

(a b c) represent statistically significant differences ($p < 0.05$) between different time points for the same group at the same location.
